# scInTime: A Computational Method Leveraging Single-Cell Trajectory and Gene Regulatory Networks to Identify Master Regulators of Cellular Differentiation

**DOI:** 10.3390/genes13020371

**Published:** 2022-02-18

**Authors:** Qian Xu, Guanxun Li, Daniel Osorio, Yan Zhong, Yongjian Yang, Yu-Te Lin, Xiuren Zhang, James J. Cai

**Affiliations:** 1 Department of Veterinary Integrative Biosciences, Texas A&M University, College Station, TX 77843, USA; qianxu0517@tamu.edu; 2 Department of Statistics, Texas A&M University, College Station, TX 77843, USA; guanxun@stat.tamu.edu; 3 Department of Oncology, Institutes of Livestrong Cancer, Dell Medical School, University of Texas at Austin, Austin, TX 78701, USA; dcosorioh@utexas.edu; 4 Key Laboratory of Advanced Theory and Application in Statistics and Data Science-MOE, School of Statistics, East China Normal University, Shanghai 200062, China; yzhong@fem.ecnu.edu.cn; 5 Department of Electrical and Computer Engineering, Texas A&M University, College Station, TX 77843, USA; yjyang027@tamu.edu; 6 Graduate Institute of Biomedical Electronics and Bioinformatics, National Taiwan University, Taipei 10617, Taiwan; f06945036@ntu.edu.tw; 7 Department of Biochemistry & Biophysics, Texas A&M University, College Station, TX 77843, USA; xiuren.zhang@tamu.edu

**Keywords:** single-cell RNA sequencing, master regulator, trajectory inference, pseudotime analysis, gene regulatory network, time-resolved data

## Abstract

Trajectory inference (TI) or pseudotime analysis has dramatically extended the analytical framework of single-cell RNA-seq data, allowing regulatory genes contributing to cell differentiation and those involved in various dynamic cellular processes to be identified. However, most TI analysis procedures deal with individual genes independently while overlooking the regulatory relations between genes. Integrating information from gene regulatory networks (GRNs) at different pseudotime points may lead to more interpretable TI results. To this end, we introduce scInTime—an unsupervised machine learning framework coupling inferred trajectory with single-cell GRNs (scGRNs) to identify master regulatory genes. We validated the performance of our method by analyzing multiple scRNA-seq data sets. In each of the cases, top-ranking genes predicted by scInTime supported their functional relevance with corresponding signaling pathways, in line with the results of available functional studies. Overall results demonstrated that scInTime is a powerful tool to exploit pseudotime-series scGRNs, allowing for a clear interpretation of TI results toward more significant biological insights.

## 1. Introduction

Single-cell RNA sequencing (scRNA-seq) has revolutionized modern biology by allowing researchers to explore cellular dynamic processes at unprecedented resolution. Indeed, scRNA-seq data may be viewed as a snapshot of the transcriptome of thousands of individual cells in a population. Each cell is at a distinct stage of the dynamic differentiation process. These dynamic processes can be studied computationally using trajectory inference (TI) approaches, also known as pseudotime analyses. Cells are ordered along a computationally-defined trajectory based on their expression profile similarity [1]. These algorithms assign each cell a pseudotime, a numeric number in arbitrary units that denotes a cell’s position during the dynamic process of interest. When a sample contains cells spanning a broad spectrum of differentiation stages, such as from a progenitor to many differentiated cellular states, computation-based TI can help gain transcriptomic insights into these complex processes. Thanks to these breakthroughs, researchers may now investigate intricate differentiation patterns and infer dynamic processes without collecting time-resolved data.

Despite these remarkable advances, most existing TI methods process each gene independently to assert a gene’s association with pseudotime, overlooking the regulatory relationships between genes, which may vary at different time points. We suggest that the information from gene regulatory networks (GRNs) should not be ignored when TI methods are used to identify master regulators that play a role in cell differentiation. Instead, single-cell GRNs (scGRNs), which hold crucial information for understanding how coordinated transcriptional patterns are associated with dynamic events, should be considered. In the existing framework of TI, differential expression (DE) analysis of individual genes is frequently adopted to compare cells with different pseudotime along the inferred trajectory. The continuous expression resolution provided by TI methods and the pseudo temporal ordering of cells along lineages is not sufficiently used in such discontinuous DE approaches.

Several approaches have been described to improve trajectory-based DE analysis by modeling gene expression as a smooth function of pseudotime along lineages. Monocle [2] and TSCAN [3] use a generalized linear model to determine whether a gene has time-dependent expression and then statistically test the significance of the regression coefficient. Other methods, such as Monocle 2 [4] and GPfates [5], allow users to test whether differences in gene expression are associated with particular branching or bifurcation events on the trajectory. TradeSeq [6] is another trajectory-based differential expression analysis that can help analyze the DE pattern between lineages. These trajectory-based approaches outperform discrete cluster-based or time-series DE analysis methods by utilizing continuous expression resolution along the trajectory. However, these methods solely assess the expression changes of a single gene along the trajectory, ignoring the impact of the gene regulatory network structure change. For this reason, the development of new strategies to identify biological processes changing along the trajectory and allow a straightforward interpretation of functional gene sets is critical for a better understanding of cellular developmental processes.

We introduce scInTime, an unsupervised machine learning framework incorporating scGRNs and user-provided pseudotime, to identify master regulators. scInTime is a versatile framework that may be used with any TI approach. There are four major steps in scInTime. (1) Trajectory inference: We first estimate the pseudotime for each cell by using a pseudotime analysis tool, such as Monocle [2]. (2) scGRN construction: For cells of different pseudotime groups, we build GRNs using principal component (PC) regression. (3) Generation of the regression coefficient matrix: With the pseudotime-series GRNs, we perform a linear regression to regress the interaction level for each pair of genes as a function of pseudotime. The regression coefficients are used to measure the trend of change of interaction strength of gene pairs across different stages of pseudotime. By collecting the regression coefficients of all pairs of genes, we obtain a regression coefficient matrix that reflects the global profile of gene regulations. (4) Analysis of the regression coefficients matrix: To aid in identifying regulatory genes, we employ a dimensional reduction method, such as tSNE, to embed genes into a lower-dimensional space to cluster genes according to their features in the regression coefficient matrix.

We validate our framework by analyzing three published scRNA-seq data sets. All data sets are time-resolved, which means they contain cells collected from different time points experimentally, so the “ground truth” of cell differentiation stages is known. With these time-resolved data sets, our results based on pseudotime are compared and validated. Data set 1 is from the differentiating neurons of the zebrafish hindbrain [7]. Data set 2 is from the human SCC6 cell line treated with cetuximab and PBS (control) [8]. Data set 3 is from differentiating mouse cardiomyocytes across fifteen real timepoints [9]. In all three cases, we set out to examine whether the top-ranking gene candidates reported by scInTime will be aligned well with those obtained from functional studies, in which corresponding master regulators are reported. If they do, then these case studies will highlight the interpretability of scInTime’s results, which may lead to meaningful explanations on how a certain pseudotime trajectory is shaped by the gene expression programs driven by the identified regulators and thus enhance the interpretation of TI results.

## 2. Materials and Methods

### 2.1. Data Sets

We obtained three published scRNA-seq data sets and used them to demonstrate the power of the scInTime framework. Data set 1: Zebrafish hindbrain data from the study of [7]. The data set was downloaded from the Broad Institute Single Cell Portal under the project SCP667. This is an aggregated data set, including three different developmental stages of zebrafish embryos, namely 16, 24, and 44 h post-fertilization (hpf). Six cell types were annotated: progenitor cells, dorsal progenitor (DP) cells, ventral-medial progenitor (VMP) cells, differentiating progenitor cells, immature neurons, and hindbrain neurons. Data set 2: Head and neck squamous cell carcinoma (HNSCC) data from the study of [8]. The data set can be found on the GEO database using accession GSE137524. Data set 3: Cardiomyocyte data set from the study of [9]. The data were downloaded from the GEO database using accession GSE164591. Data from cardiomyocytes collected at the time ranging from embryonic day (e)14 to postnatal day (p)84 were used in the analysis. For all data sets, cells with fewer than 1000 UMI counts or more than 10 percent mitochondrial reads (mt-DNA%) were excluded from the downstream analyses. Data normalization, scaling, principal component analysis, and visualization were carried out using the Seurat package v.4.0.2 [10].

### 2.2. Pseudotime Determination for Cells

Monocle 3 [11] was used to perform pseudotime analysis. The input data were normalized using the preprocess_cds function. The align_cds function was then used to eliminate batch effects from the zebrafish hindbrain data and the mouse cardiomyocytes data, both of which contain numerous sampling timepoints (num_dim = 50, alignment group = “library” or “stage”). After that, UMAP [12] dimensional reduction (reduce_dimension: umap.min_dist = 0.2) and cell clustering (cluster_cells function) were applied to the data. A principal graph was learned from previously reduced dimensions and was visualized through the UMAP using the learn_graph function, representing the trajectory through development. The graph was, in turn, used to order cells through the developmental program (order_cells function) as pseudotime using cells expressing selected markers as the trajectory root cells. The selective markers to differentiate the root cells are described in the main text above. We used the Correlation of Connectome and Transcriptome (CCAT) method [13] to estimate the differentiation potency for each cell.

### 2.3. Time-Series scGRN Construction and Generate the Regression Coefficient Matrix

After performing quality control and Pearson residuals normalization [14] for the raw data, we ordered cells and separated them into 10 groups using the pseudotime obtained in the previous step. The overlap between groups was allowed in this case to ensure that each group had at least 500 cells. We also calculated the average pseudotime for each group of cells and normalized them into (0,1) and denoted them as $t_{i}$, where i=1, 2, …, 10. To build the network for each group of cells, we utilized PC regression, an approach we adopted previously in developing scTenifoldNet [15].

Let $X^{i}\in {\mathbb{R}}^{p\times n\;}$ be the i-th subgroup scRNA-seq data matrix that reflects gene expression levels for *p* genes in *n* cells. We then construct a GRN with an adjacency matrix $W^{i}$ for each $X^{i}$ using PC regression. Each time, we pick one gene as the response gene and use all the other genes as explanatory variables. We first apply a PC analysis to the explanatory variables, then regress the response variable on d leading PCs (*d* << *n*). Next, we transform the regression coefficients of d leading PCs into the coefficients of original explanatory variables, which reflect the interaction strengths between the i-th gene and the other genes. By processing the above PC regression for p times using different genes as the response, we finally combine the coefficients of p regression models into a p×p  adjacency matrix $W^{i}$, where the (i, j) entry records the regression coefficient of the i-th gene on the j-th gene. Thus, $W^{i}$ collects the strength of interactions between each pair of genes.

The next step is to perform linear regression on the normalized pseudotime $t=\left(t_{1},\ldots ,\;t_{10}\right)$ by setting the adjacency matrix W as the response variable. More specifically, given any pairs of genes (i,j), let $\left(W_{ij}^{1},\;W_{ij}^{2},\;\ldots ,\;W_{ij}^{10}\right)$, the strengths of the interaction between the i-th gene and the j-th gene in the 10 subgroups, be the response. Let the corresponding pseudotime $\left(t_{1},\;t_{2},\ldots ,\;t_{10}\right)$ be the regressor; then, perform the simple linear regression. Denote the slope estimator as $\beta_{ij}$. By combining the slope estimators of the regressions of all pairs of genes, we obtain the regression coefficients matrix $\beta \in \;{\mathbb{R}}^{p\times p\;}$ with the (i,j) entry is $\beta_{ij}$.

### 2.4. Code Availability

We provided code and data repositories to support this study. The main repository contains R code for reproducing figures and analyses presented in the paper and can be found at https://github.com/cailab-tamu/scInTime (accessed on 4 February 2022).

## 3. Results

### 3.1. The scInTime Architecture

#### 3.1.1. Construct Pseudotime-Series Gene Regulatory Networks

**Figure 1:**
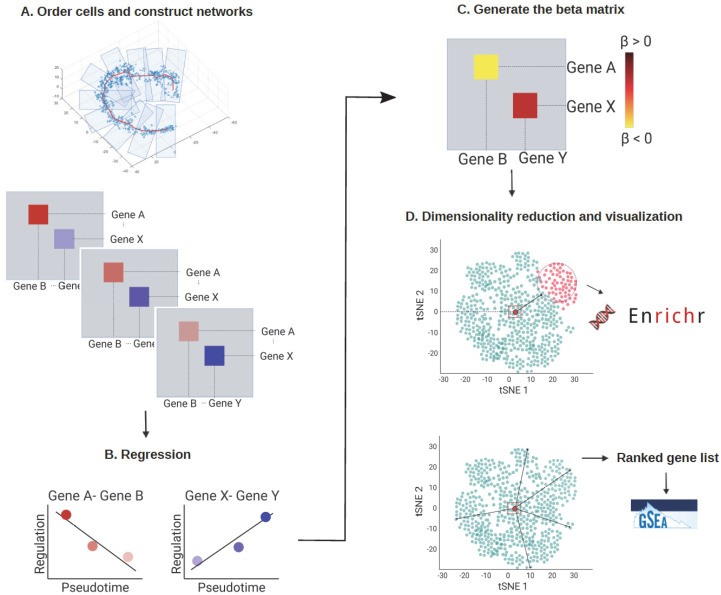
The analytical framework of scInTime. (**A**) Step 1: Order cells and construct networks. The cells are arranged and divided into 10 subgroups according to the pseudotime inferred using a pseudotime analysis tool, such as Monocle 3. Each group stands for a different transition state. Next, an scGRN is constructed for each group of cells. Each scGRN depicts gene-gene regulatory relationships at the given pseudotime point. A total of 10 scGRNs are constructed, each comprising the progressive transcriptomic profile of the given cellular process. (**B**) Step 2: Fit a linear regression model to the 10 scGRNs. We fit a linear regression model for the 10 coefficients on the state index for each pair of genes. If this pair of genes’ regulation strength increases along the 10 stages, the regression coefficient will be positive and vice versa. (**C**) Step 3: Generate the regression coefficient matrix. All the regression coefficients are collected into a single regression coefficients matrix, representing the global profile of all pairs of gene-gene regulation changes across the given cellular process. (**D**) Dimensionality reduction and visualization of the regression coefficients matrix. We used tSNE embedding to reduce dimensionality on the regression coefficients matrix and visualize it. Each dot in the tSNE plot represents a gene embedded in the 2D plot based on its global profile in the regression coefficients matrix. The k-means clustering algorithm divides the genes in the regression coefficients matrix into 50 clusters. Enrichr analysis is performed on selected gene clusters. The genes are rank ordered according to their Mahalanobis distance to the embedding center. Then, the GSEA analysis is performed on the ranked gene list.

To identify the cell stage according to their pseudotime and biological transition states, cells were ordered according to their pseudotime (e.g., estimated using Monocle 3) or their differentiation potency (e.g., estimated using CCAT) and then divided into multiple (e.g., 10) pseudotime-series subgroups corresponding to cells’ biological transition states (Figure 1A, up). Thereby, cells were assumed to be sorted according to their biological transition states, which could come from cellular processes, such as cell differentiation or cell reprogramming in response to a stimulus. After assigning pseudotime to cells and dividing cells into subgroups, we constructed scGRN for each subgroup using PC regression [15] (Figure 1A, bottom). PC regression is computationally efficient when compared to other GRN inference algorithms, such as GENIE3 [16]. In addition, PC regression constructs gene regulatory network that are weighted and directed, whereas GENIE3 only returns the associations between genes. In each scGRN, interacting genes were connected with the weight of each linker reflecting how strongly each pair of genes interacted.

#### 3.1.2. Regression Analysis

Regression analysis was then performed to concatenate all the gene regulatory information from the 10 scGRNs into one single regression coefficient matrix. We fitted a linear regression model for the 10 regulation coefficients for each pair of genes to obtain a regression coefficient. Each regression coefficient stands for the change in the interaction strength between the gene pair as a function of pseudotime.

#### 3.1.3. Build Regression Coefficients Matrix

We then collected the regression coefficient of each pair of genes to generate a single regression coefficients matrix (Figure 1C). If the regulation strength between gene i and gene j became stronger along the pseudotime, the regression coefficient value *β*(*i*, *j*) would be positive. Conversely, if the regulation strength between the gene pairs became weaker along the pseudotime, the *β*(*i*, *j*) would be negative.

#### 3.1.4. Analysis of Regression Coefficients Matrix

To visualize relationships between genes with features in the regression coefficients matrix, we used the dimension reduction method tSNE to embed genes into a 2D scatter plot (Figure 1D). Each dot in the tSNE plot represented an embedded gene whose position was determined by the gene’s profile in the regression coefficient matrix. Then, we used k-means clustering and Mahalanobis distance measurement to analyze the regression coefficients matrix.

**Figure 2:**
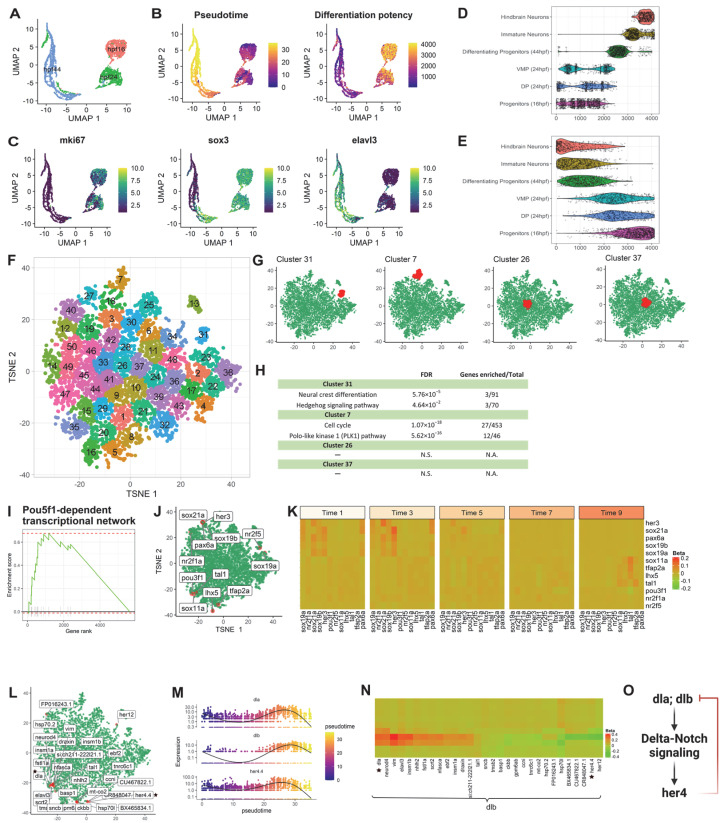
scInTime analysis with time-resolved scRNA-seq data from zebrafish hindbrain reveals gene expression programs in neurogenesis. (**A**) Visualization of cells using UMAP embeddings plot. The cells are collected at 16 hpf, 24 hpf, and 44 hpf and color-coded accordingly. (**B**) UMAP visualization of cells colored according to their pseudotime estimation (left panel) and differentiation potency (right panel). The pseudotime is estimated using Monocle 3. The level of differentiation potency is estimated using the CCAT algorithm. (**C**) Expression level (log-transformed UMIs) of three representative genes, expressed highly at three different stages of development. (**D**) Boxplot of cells in different developmental stages and their pseudotime estimates. (**E**) Boxplot of cells in different developmental stages and their differentiation potency. (**F**) Clustering of genes in the tSNE plot. The 5863 genes are clustered into 50 clusters using the k-means algorithm. Genes in the same cluster have a similar profile in the regression coefficient matrix. (**G**) The position of four clusters #13, #14, #15, and #16 in the tSNE plot. Clusters #13 and #14 are in the peripheral region of the 2D tSNE plot; clusters #15 and #16 are in the central region. (**H**) Results of enrichment analysis for the four clusters in (**G**). N.S., not significant. (**I**) GSEA enrichment plot for *Pou5f1*-dependent transcriptional network. (**J**) tSNE visualization of genes (*n* = 5863) embedded in a 2D plot based on each gene’s profile in the regression coefficient matrix. In the plot, each dot represents a gene. The leading-edge genes for the *Pou5f1*-dependent transcriptional network given in GSEA analysis are highlighted red. (**K**) A pseudotime-series heatmap showing the change of regulatory strength between genes as a function of time. The genes shown are the leading-edge genes in the *Pou5f1*-dependent transcriptional network, as in (**I**,**J**). (**L**) Positions of 30 genes with the highest regulatory strength with *dlb* in the tSNE plot. The genes labeled with black stars are from the model showed in (**O**), as in (**N**). (**M**) The expression level of three selected genes (*dla, dlb*, and *her4.4*) as a function of pseudotime shows a strong covarying relationship among them. None of these genes’ expressions show a monotonic relationship with pseudotime. (**N**) Heatmap of regression coefficients between *dlb* and each of its top 30 regulated genes across 10 pseudotime intervals. The genes labeled with black stars are from the model showed in (**O**), as in (**L**,**O**) Model of gene regulation: Products of genes *dta* and *dtb* activate genes in the delta-notch signaling pathway and then *her4*, which is involved in a negatively regulatory feedback loop suppressing *dlb* expression.

We used the k-means clustering algorithm to divide the genes into 50 clusters (Figure 2F) to explore and compare the genes’ features on the tSNE embedding. Next, Enrichr [17] was used to perform the gene set enrichment analysis for each group of genes (Figure 1D, up). This systematic screen revealed many known and unexpected biological pathways associated with the genes in each cluster (Figure 2G,H). In addition, one feature was noteworthy: each gene’s relative position in the tSNE embedding plot was correlated with the gene’s feature in the regression coefficients matrix. More specifically, the clusters located in the peripheral region of the tSNE plot tended to contain genes with more biologically meaningful functions, and the enrichment results were more statistically significant (Figure 2H). We also found that the most highly variable genes (HVGs) calculated using the Seurat package [10] were mainly located in the peripheral region of the tSNE plot (Figure S1A). Less variable genes, by contrast, were more likely to be situated in the center of the plot. Because each regression coefficient value in the regression coefficient matrix stands for the changes of each gene’s regulation strength across the pseudotime, a possible reason was that genes with a dynamical regulatory relationship with other genes would have a greater absolute regression coefficient value. As a result, these genes would be pushed to the peripheral region of the tSNE embedding. Other genes with little or no regulatory relationship across the pseudotime, by contrast, would have a small absolute regression coefficient value and stay at the center of the tSNE plot.

For the Mahalanobis distance measurement, we ranked all the genes in the tSNE embedding by their Mahalanobis distance to the center of the plot (Figure 1D, up), then tested if the location of each cluster on the tSNE embedding was associated with any biologically meaningful functions, thus resulting in a ranked gene list. We then applied gene set enrichment analysis (GSEA) to reveal the biological processes enriched in this ranked gene list (Figure 1D, bottom). Indeed, genes with relevant functions were likely to be toward the top of the gene list, suggesting that the genes at the peripheral region of the tSNE plot might recapitulate the most important regulators related to the cellular process in question.

### 3.2. Applications to Time-Resolved scRNA-seq Data

#### 3.2.1. Application 1: Zebrafish Hindbrain

To investigate the applicability of the pipeline, we first performed scInTime analysis on a published data set from the zebrafish hindbrain to identify the essential regulatory genes associated with the neurogenesis process [7]. This data set consists of 4112 single cells spanning three developmental stages of neuronal differentiation (Figure 2A): 16 hpf (the beginning of neurogenesis), 24 hpf (early stages of development), and 44 hpf (pattern of neurogenic zones fully established). We used Monocle 3 to assign each cell a pseudotime according to their position in the differentiation trajectory (Figure 2B, left). We also used the CCAT package to estimates the cells’ differentiation potency as a complementary approach to Monocle 3 (Figure 2B, right). Not surprisingly, we found that the progenitor cells from 16 hpf, dorsal progenitors from 24 hpf, and ventral-medial progenitors from 24 hpf have relatively lower pseudotime and high differentiation potency. The differentiating progenitors from 44 hpf, immature neurons, and hindbrain neurons, on the contrary, have relatively high pseudotime and low differentiation potency (Figure 2D,E). We also showed the expression level of three marker genes expressed during neurogenesis (Figure 2C). The *mki67* is the proliferation and immature neuron marker, *sox3* is the intermediate differentiation state marker, and *elavl3* is a neuronal marker.

Next, the cells (*n* = 4112) were divided into 10 subgroups according to their pseudotime. Each subgroup contained approximately 400 cells (Group 1: 1:400; Group 2: 401:800; and so on). We filtered out the genes expressed by fewer than a certain number of cells (e.g., 5% of all the cells) and kept 5863 genes for the downstream analysis. After the scGRNs construction and regression, we obtained a 5863-by-5863 regression coefficient matrix. We then performed dimensionality reduction using the tSNE (Figure S1A). We calculated the HVGs (*n* = 2000) in this data set using the Seurat package and highlighted the HVGs in the tSNE embedding (Figure S1A). The HVGs locate favorably to the peripheral region on the tSNE plot. We also used another dimensionality reduction method, PHATE [18], to create a new embedding on the regression coefficient matrix. We highlighted the HVGs in the PHATE embedding (Figure S1B) and found that the HVGs were primarily located in the most distinct endpoints. In contrast, most non-HVGs were situated in the middle of each PHATE branch, suggesting that the HVGs shared strong concordance with the most distinct genes detected by PHATE. These results showed that both tSNE and PHATE tend to put data points (in our case, genes) with characteristic features (in our case, more variable expression) at peripheral embedding regions. This embedding pattern of genes inspired us to use tSNE and PHATE to embed genes from the regression coefficient matrix.

Therefore, with the regression coefficient matrix, we embedded genes with tSNE and then clustered them into 50 clusters based on their tSNE embedding (Figure 2F). In the PHATE embedding, we randomly selected multiple clusters from the tSNE embedding and highlighted the genes from each cluster (Figure S1C). We observed that genes in the same tSNE cluster are either centered together at the middle of the PHATE embedding or located at the same branch, indicating that tSNE and PHATE performed similarly in identifying distinct gene sets. Furthermore, in the regression coefficient matrix, genes found close to each other on the tSNE embedding share a similar global regulatory relationship with other genes.

Having confirmed that the lower dimensional tSNE embedding can reveal the underlying structure of the regression coefficient matrix, we next investigated the collective functions in different tSNE clusters of genes. To address this, we applied the Enrichr to perform enrichment analysis for genes in selected clusters (Figure 2G,H). We found that clusters #31 and #7, which are located at the peripheral region of the tSNE plot, are enriched in neurogenesis associated pathways, such as neural crest differentiation, hedgehog signaling pathway, cell cycle, and Polo-like kinase 1 (PLK1) pathway. The clusters located at the center of the tSNE plot, e.g., clusters #26 and #37, have no statistically significant pathway enriched.

We identified 272 pathways with false discovery rate (FDR) < 0.05 after ranking-ordering all the genes based on their Mahalanobis distance to the center of the tSNE plot and performing the GSEA (Table S1, Figure 2I–K), including regeneration of zebrafish CNS, adult neurogenesis, delta-Notch signaling, lateral inhibition in zebrafish spinal cord development, and so on. These enriched gene sets revealed crucial genes and pathways associated with zebrafish neurogenesis and hindbrain patterning. Figure 2I,J show the enrichment score and the tSNE embedding of the leading-edge genes from the zebrafish *Pou5f1*-dependent transcriptional networks in temporal control of the early development pathway. The heatmap from Figure 2K reveals the involvement of two sets of genes during the early (*her3* [19]; *sox19a* [20]; *sox19b* [21]; *sox21a*) and late (*tal1; tfap2a* [7]) processes of neural development.

scInTime can discover developmental master regulators and their regulation relationships regardless of their expression level (Figure 2L–O). For example, newly specified neurons express Delta genes (i.e., *dla* and *dlb*) to promote Notch activity in neighboring neuron precursors to maintain a proliferative stem cell population [22]. The transcription factor *her4* is a downstream effector of Notch-Delta signaling [23]. Activation of *notch1* leads to strong activation of *her4*. Moreover, *her4* is involved in a negatively regulatory feedback loop to suppress *dlb* expression [24]. Since *dlb* is highly expressed in neuronal cells, we choose *dlb* to study its regulation across the pseudotime. Figure 2L shows the tSNE embedding of the top 30 regulators of the *dlb*, with the greatest absolute regression coefficient values. Figure 2M shows the dynamic expression level of the three genes, *dla, dlb* and *her4.4*. We showed the regulation changes between *dlb* and its top 30 regulators in 10 pseudotime stages (Figure 2N). We observed a gene regulation model involving *dla*, *dlb*, and *her4.4*, in which *dla* and *dlb* activate *her4.4* through the Notch-delta signaling pathway. *Her4.4*, on the contrary, can inhibit the expression of *dla* and *dlb*. It is worth noting that only *dla* and *dlb* were identified as DE genes using Monocle 3, whereas *her4.4* was not identified as differentially expressed gene. Thus, scInTime can extend beyond traditional DE analysis by discovering developmental regulators and inferring their gene regulatory relationships across the pseudotime regardless of their expression level.

#### 3.2.2. Application 2: HNSCC Cell Line

Patients with head and neck squamous cell carcinoma (HNSCC) benefit from cetuximab treatment which targets the epidermal growth factor receptor (EGFR). However, resistance to cetuximab occurs clinically early on. To overcome this unique resistance mechanism, researchers must learn more about the critical gene regulators and their regulation relationships during cetuximab treatment.

**Figure 3:**
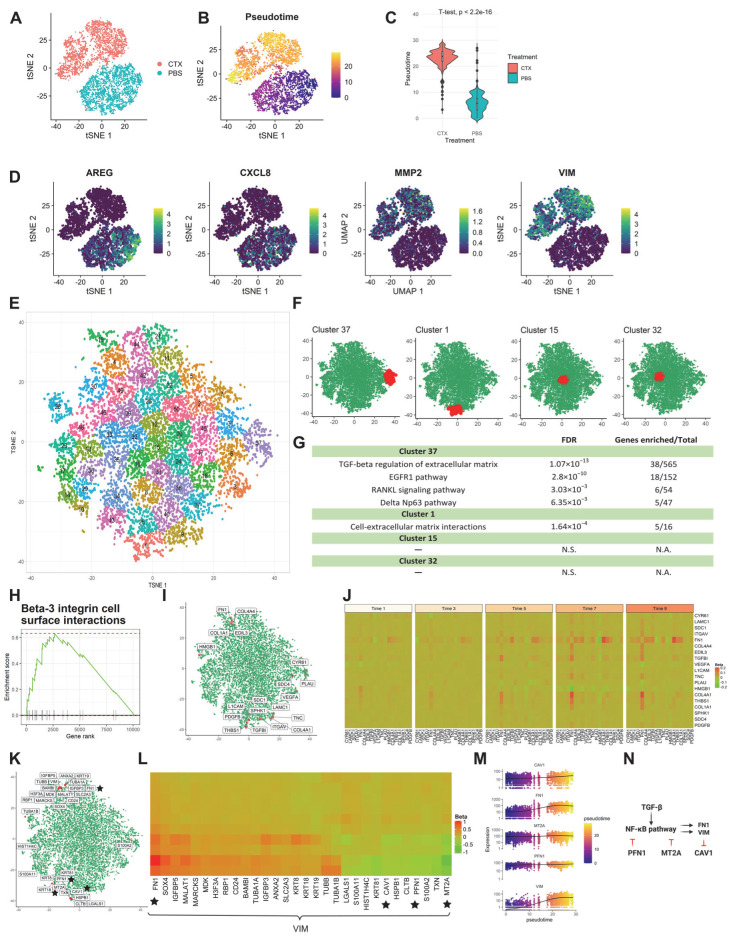
scInTime analysis with scRNA-seq data from HNSCC cell line reveals gene expression programs in cetuximab resistance development. (**A**) Visualization of cells using tSNE embeddings plot. The cell line received cetuximab (treated) or PBS (untreated), and after five consecutive days, the cells were collected for scRNA-seq and color-coded accordingly. (**B**) Two-dimensional representation of the data using tSNE. The trajectory inferred by Monocle 3 is displayed. (**C**) Boxplot of cells in different treatment groups and their pseudotime estimates. (**D**) Expression level (log-transformed UMIs) of four representative genes, expressed differentially at two different treatments. (**E**) Clustering of genes in the tSNE plot. In the plot, each dot represents a gene. The 10,378 genes are clustered into 50 clusters using the k-means algorithm. Genes in the same cluster have a similar profile in the regression coefficient matrix. (**F**) Positions of four clusters, #37, #1, #15, and #32, in the tSNE plot. Clusters #37 and #1 are in the peripheral region of the 2-D tSNE plot; clusters #15 and #32 are in the central region. (**G**) Results of enrichment analysis for the four clusters in (**G**). N.S., not significant. (**H**) GSEA enrichment plot for Beta-3 integrin cell surface interactions. (**I**) tSNE visualization of genes (*n* = 10,378) embedded in a 2D plot based on each gene’s profile in the regression coefficient matrix. The leading-edge genes for Beta-3 integrin cell surface interactions given in GSEA analysis are highlighted red. (**J**) A pseudotime-series heatmap showing the change of regulatory strength between genes as a function of time. Genes shown are the leading-edge genes in Beta-3 integrin cell surface interactions, as in (**I**). (**K**) Position of 30 genes with highest regulatory strength with *VIM* in the tSNE plot. The genes labeled with black stars are from the model showed in (**N**), as in (**L**). (**L**) Heatmap of regression coefficient values (levels of regulatory strength) between *dlb* and each of its top 30 regulated genes across 10 pseudotime intervals. The genes labeled with black stars are from the model showed in (**N**), as in (**K**). (**M**) The expression level of three selected genes (*CAV1, MT2A,* and *PFN1*) as a function of pseudotime, showing a strong covarying relationship among them. None of these genes’ expressions show a monotonic relationship with pseudotime. (**N**) The model of the regulations among the selected genes. The activation of the NF-κB signaling pathway will upregulate the transcription of *VIM. VIM* unidirectionally down-regulates *CAV1. PFN1* and *MT2A* inhibit *VIM* expression by inhibiting the activation of the NF-κB signaling pathway.

To determine whether scInTime can provide insights into the potential drivers of the trajectory during the cetuximab resistance development, we analyzed SCC6 cells from another published data set [8]. This data set contains the SCC6 cell line and was treated with cetuximab or PBS (untreated controls) for five consecutive days. After removing low-quality cells and applying principal component analysis to visualize the data, we confirmed two clusters of cells: cetuximab-treated (*n* = 2703) and PBS-treated (*n* = 2626) cells (Figure 3A). To trace the cells’ response to cetuximab treatment, we combined the two groups of cells as a whole and used Monocle 3 to construct a trajectory involving all the cells (*n* = 5369). The trajectory starts from the PBS-treated sample and ends at the cetuximab-treated sample. Each cell was assigned a pseudotime accordingly (Figure 2C). *t*-test showed a significant difference in the pseudotime between the two groups (Figure 2C), suggesting that the cells have progressed to a different state with altered transcriptome to overcome the EGFR inhibition. We also found four marker genes with divergent expression patterns prompted by cetuximab: *AREG* and *CXCL8* were down-regulated by cetuximab, whereas *MMP2* and *VIM*, two epithelial-to-mesenchymal transition (EMT) markers, were upregulated. These results align well with earlier studies [25,26], suggesting that the cetuximab might have promoted EMT in the SCC6 cell line.

The cells were divided into 10 subgroups as described previously. After the scGRNs construction and regression, we obtained a 10,378-by-10,378 regression coefficient matrix. The tSNE was used to embed the genes and k-means clustering was used to cluster the genes into 50 clusters (Figure 3E). Enrichr analysis revealed several biological functions of the peripheral clusters #37 and #1, including TGF-beta regulation of extracellular matrix, EGFR1 pathway, RANKL signaling pathway, delta Np63 pathway, and cell-extracellular matrix interactions. In contrast, the center clusters #15 and #32 have no biological pathways significantly enriched (Figure 3F,G).

GSEA analysis of the resulting ranked gene list also revealed a series of biological functions that are altered during cetuximab treatment. One example is the Beta-3 integrin cell surface interactions pathway (Figure 3H–J). Integrins are the most common cellular adhesion receptors and are implicated in nearly every stage of cancer development [27]. In pancreatic cancer cells, overexpression of integrin β1 activates the FAK/tyrosine kinase/Akt pathway, resulting in EGFR-independent cell proliferation and, as a result, the ability to overcome EGFR suppression by cetuximab [28].

GSEA analysis of the ranked gene list also revealed other adaptive mechanisms induced by cetuximab treatment (Table S2), including RANKL regulation of apoptosis and immune response, delta Np63 pathway, and AP-1 transcription factor network. In gastric cancer cells, the RANKL/RANK pathway can abrogate cetuximab sensitivity by phosphorylation of EGFR, AKT, and ERK [29], whereas ΔNp63α expression can increase EGFR mRNA, total EGFR protein, and phospho-EGFR (Y1086), thus contributing to tumor cell proliferation [30,31]. TGF induction of AP-1 proteins was also highly potentiated by P63 and EGFR. [32]. The pathways identified by scInTime analysis confirmed the cooperative pro-oncogenic functions of EGFR, AP-1, p63, and TGFβ, suggesting an early onset of cetuximab resistance driven by these adaptive mechanisms.

scInTime successfully recapitulated the crucial regulators in the NF-kB signaling pathway, which is known to be responsible for resistance to cetuximab therapy [33] and regulates EMT genes [32]. The NF-κB signaling pathway is tightly regulated by an inhibitory molecule, namely IκBα/β/γ (*IκB*). Upstream activation like the TGF-β signaling pathway can release the NF-κB dimer from IκB, thus activating the downstream target genes like vimentin (*VIM*) [34] and fibronectin (*FN1*) [35] to promote the EMT process. Profilin (*PFN1*), in contrast, can inhibit NF-κB activation by inhibiting IKK phosphorylation [36]. Another inhibitory gene, metallothionein 2A (*MT2A*), can inhibit the NF-κB pathway by upregulating IκB-α and down-regulating p-IκB-α [37,38]. Vimentin can unidirectionally (down)-regulate caveolin-1 (*CAV1*) [39]. In scInTime analysis, we not only identified the genes associated with TGF-beta regulation of extracellular matrix pathway in peripheral clusters in the tSNE embedding (Figure 3F,G) but also revealed a gene regulation model during this EMT process by analyzing the top 30 regulators of the EMT marker gene, *VIM* (Figure 3K–N). In this model, the *VIM* gene is negatively associated with *PFN1, MT2A,* and *CAV,* and positively related with *FN1*, aligning well with the previous studies mentioned above. Note that although the fit_models function in the Monocle 3 package detected all five genes in this model as genes that change as a function of pseudotime (Figure 3M), Monocle 3 did not consider the corresponding regulatory relationship among those genes.

#### 3.2.3. Application 3: Mouse Cardiomyocytes

**Figure 4:**
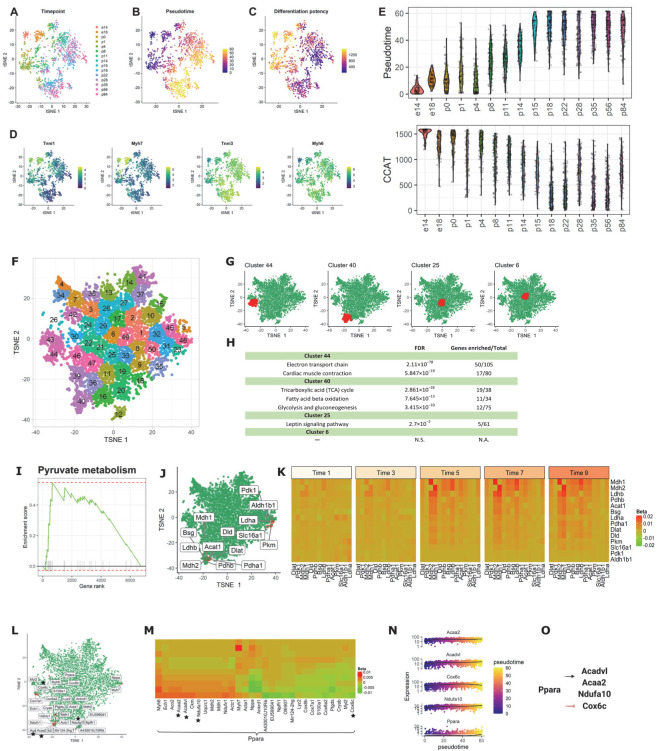
scInTime analysis with time-resolved scRNA-seq data from mouse CMs reveals gene expression programs in CM maturation. (**A**) Visualization of cells using tSNE embeddings plot. The cells are collected at 15 time points and color-coded accordingly. (**B**) tSNE visualization of cells colored according to their pseudotime estimation. The pseudotime is estimated using Monocle 3. (**C**) tSNE visualization of cells colored according to their differentiation potency. The differentiation potency is estimated using CCAT. (**D**) Expression level (log-transformed UMIs) of four representative genes, expressed differentially at different time points. (**E**) Boxplot of cells in different developmental stages (time points), colored by their pseudotime (up) and differentiation potency (bottom). (**F**) Clustering of genes in the tSNE plot. In the plot, each dot represents a gene. The 6969 genes are clustered into 50 clusters using the k-means algorithm. Genes in the same cluster have a similar profile in the regression coefficient matrix. (**G**) Positions of four clusters: #44, #40, #6, and #25, in the tSNE plot. Clusters #44 and #40 are located in the peripheral region of the 2D tSNE plot; clusters #6 and #25 are located in the central region. (**H**) Results of enrichment analysis for the four clusters in (F). N.S., not significant; N.A., not applicable. (**I**) GSEA enrichment plot for Pyruvate metabolism. (**J**) tSNE visualization of genes (*n* = 6969) embedded in a 2D plot based on each gene’s profile in the regression coefficient matrix. The leading-edge genes for pyruvate metabolism given in GSEA analysis are highlighted in red. (**K**) A pseudotime-series heatmap showing the change of regulatory strength between genes as a function of time. The genes shown are the leading-edge genes in Pyruvate metabolism, as in (**I**,**J**). (**L**) Positions of the 30 genes with the highest regulatory strength with *Ppara* in the tSNE plot. The genes labeled with black stars are from the model showed in (**O**), as in (**M**). (**M**) Heatmap of regression coefficient values (levels of regulatory strength) between *Ppara* and each of its top 30 regulated genes across 10 pseudotime intervals. The genes labeled with black stars are from the model showed in (**O**), as in (**L**). (**N**) The expression level of three selected genes (*Acaa2, Acadvl*, and *Ndufa10*) as a function of pseudotime. (**O**) The model of the regulations among the selected genes. *Ppara*, as an important regulator of fatty acid metabolism, activates fatty acid metabolism genes, including *Acadvl* and *Acaa2*, and genes related to mitochondrial energy metabolism (*Ndufa*). *Ppara* agonists can inhibit *Cox* genes expression.

To further evaluate scInTime’s ability to identify the critical genes associated with stem cells and differentiation, we analyzed another published time-resolved data set from mouse cardiomyocytes (CMs). This data set contains a total of 1596 left ventricular free wall CMs spanning 15 timepoints ranging from embryonic day (e)14 to postnatal day (p)84 (Figure 4A). To understand the gradual transcriptomic changes over the CM maturation process, we first performed trajectory inference using Monocle 3 and stemness estimation using CCAT (Figure 4B,C). We also plotted the Monocle 3-computed pseudotimes, and CCAT estimated differentiation potency at each biological timepoint, which validated the progressively increasing pseudotime (Figure 4E, top) and decreasing CCAT score (Figure 4E, bottom) over biological time. Additionally, feature plot of the four marker genes (Figure 4D) also uncovered the sarcomeric isoform switching from fetal myosin heavy chain 7 (*Myh7*) to adult *Myh6* as well as the switching from slow skeletal troponin I (*Tnni1*) to cardiac troponin I (*Tnni3*) in mature CMs, matching earlier reports about rodents’ CM maturation process [40,41,42,43].

After the scGRNs construction and regression, we obtained a 6969-by-6969 regression coefficient matrix for downstream analysis. We first embedded the genes in the regression coefficient matrix using tSNE; then, we clustered the genes into 50 clusters (Figure 4F). We observed a similar pattern described above: the marginal clusters showed more statistically significant pathways associated with the CM maturation process compared with the central clusters (e.g., terms related to cardiac structure, contractile function, electrophysiology, and metabolism). For example, the genes in clusters #44 and #40 are enriched in the electron transport chain, cardiac muscle contraction, tricarboxylic acid cycle, fatty acid beta-oxidation, and glycolysis and gluconeogenesis (Figure 4G,H). In contrast, central clusters, e.g., clusters #25 and #6, do not have CM maturation-related pathways significantly enriched.

Having assessed the functions on individual clusters, we next asked whether the genes prioritized by scInTime can recapitulate pathways associated with CM maturation. We rank-ordered the genes in the tSNE embedding by their Mahalanobis distance to the center of the tSNE embedding. We observed several gene sets related to the CM’s morphogenesis and maturation significantly enriched in this rank-ordered gene list (Table S3), including the actin cytoskeleton regulation, the calcium regulation in the cardiac cell, and the PPAR signaling pathway. These pathways are associated with the functional features for more mature CMs. In addition, we discovered an enhanced regulation pattern in the pyruvate metabolism pathway, a marker for mitochondrial oxidative metabolism, across the pseudotime. This suggests that baseline mitochondrial respiration and respiratory capacity in CMs increased immediately after birth, consistent with previous observations on the “fetal switch” from glycolysis to oxidative glucose metabolism during CM maturation [44,45,46].

Peroxisome proliferator-activated receptors (*PPARs*) signaling regulates numerous genes in mitochondria biogenesis and cellular metabolism [47]. *Ppara* is a PPAR family transcription factor found in various tissues, including hepatocytes and CMs, where fatty acids are mostly catabolized [48,49]. *Ppara* controls fatty acid metabolism and activates genes involved in fatty acid metabolism, such as *Acadvl* and *Acaa2*, as well as mitochondrial energy metabolism genes (*Ndufa*). In addition, several *Ppara* agonists, such as Wy-14643 [50,51] and LYSO-7 [51], can inhibit *Cox* genes expression [52], implying a negatively regulation mechanism between these two genes. To gain mechanistic insights into the regulators governing postnatal CM maturation, we looked at the top 30 genes that have the strongest regulatory relationships with *Ppara*. Using scInTime analysis, we recovered several fatty acid metabolism genes and several *cox* genes mentioned above. Furthermore, we discovered a gene regulatory model incorporating these genes, in which *Ppara* upregulates *Acadvl, Acaa2,* and *Ndufa* while down-regulating *Cox* genes, such as the *Cox6c* gene.

## 4. Discussion

This work proposes scInTime, an unsupervised machine learning framework for studying cell differentiation, incorporating scGRNs and pseudotime information. This flexible framework allows researchers to identify master regulators and underlying regulatory programs that shape the trajectory inferred by a TI method. Unlike other methods that perform differential expression analyses with cells with different pseudotime along the trajectory, scInTime incorporates scGRNs with dynamic interaction levels between gene pairs to detect the gene regulation relationships that evolve over pseudotime.

The flexibility provided by scInTime is crucial, as various TI methods based on different strategies may produce different trajectories even for the same given scRNA-seq data set. scInTime can be utilized as an effective “third-party” tool, independent from any TI methods, to provide in-depth downstream analysis, complementing existing TI methods and extending the function of those TI methods.

We demonstrated scInTime’s ability to find master regulators in three different cellular developmental systems with these considerations. By evaluating these published data sets, we showcased the interpretability of the scInTime framework. As said, we demonstrated that scInTime could be used as the last step in any downstream analysis of time-course studies. To the best of our knowledge, scInTime is the first tool with such a GRN-based TI interpretation framework. We therefore anticipate that scInTime as a powerful and flexible computational method, extending the function of TI inference, will contribute to the detection of mater regulators of cell differentiation in various cellular systems studied in the future.

## Data Availability

Data set 1: Zebrafish hindbrain data were downloaded from the Broad Institute Single Cell Portal (https://singlecell.broadinstitute.org/, accessed on 14 June 2021) under the project SCP667. Data set 2: Head and neck squamous cell carcinoma data were downloaded from the GEO database using accession GSE137524. Data set 3: Cardiomyocyte data set was downloaded from the GEO database using accession GSE164591.

## Supplementary Materials

The following supporting information can be downloaded at: https://www.mdpi.com/article/10.3390/genes13020371/s1, Figure S1: Visualization of the regression coefficient matrix on tSNE and PHATE embedding. (**A**) tSNE embedding of the regression coefficient matrix (2000 HVGs are highlighted in red). (**B**) PHATE embedding of the regression coefficient matrix (the 2000 HVGs are highlighted in red). (**C**) Visualization of four selected clusters on the PHATE embedding. Genes of each cluster are highlighted in red. Table S1: Results of GSEA analysis of the ranked gene list from scInTime analysis of the zebrafish hindbrain data. Table S2: Results of GSEA analysis of the ranked gene list from scInTime analysis of the HNSCC data. Table S3: Results of GSEA analysis of the ranked gene list from scInTime analysis of the mouse CM data.
