# Supplementary material for: scInTime: A Computational Method Leveraging Single-Cell Trajectory and Gene Regulatory Networks to Identify Master Regulators of Cellular Differentiation
Source: Genes (Basel). 2022 Feb 18;13(2):371. doi: 10.3390/genes13020371 (PMC8872487; doi:10.3390/genes13020371)
Supplement: Supplementary file 1 [file genes-13-00371-s001.zip › genes-1555620.supplementary/Figure S1.pdf]

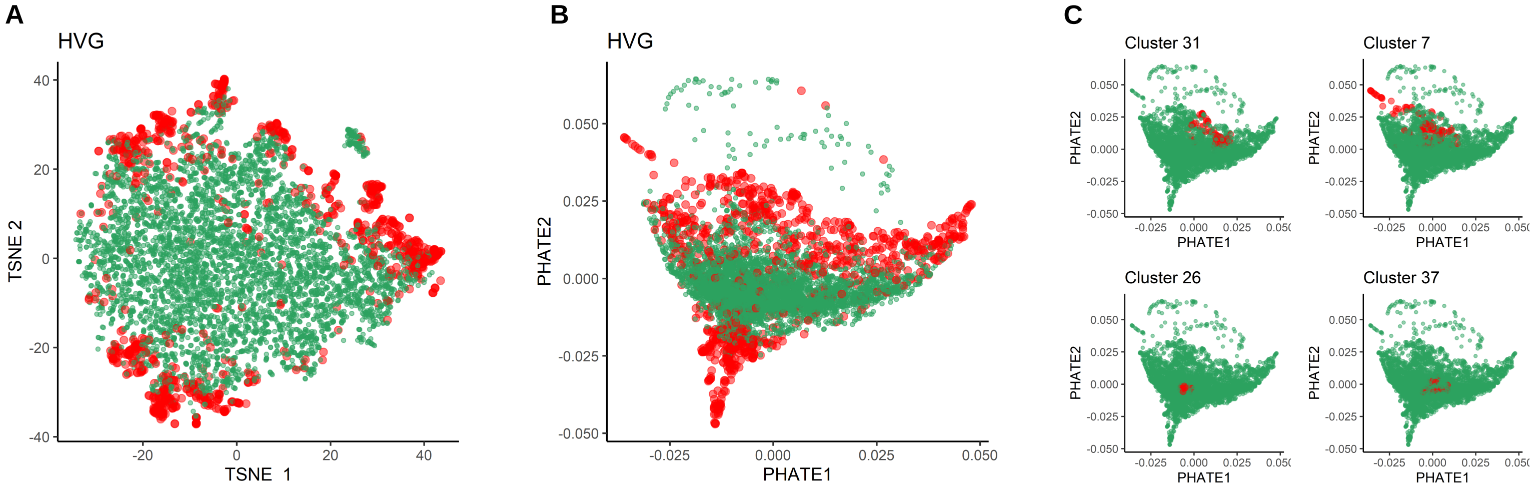

Figure S1. Visualization of the regression coefficient matrix on tSNE and PHATE embedding. (**A**) tSNE embedding of the regression coefficient matrix (2000 HVGs are highlighted in red). (**B**) PHATE embedding of the regression coefficient matrix (the 2000 HVGs are highlighted in red). (**C**) Visualization of four selected clusters on the PHATE embedding. Genes of each cluster are highlighted in red.
